# Impact of dentofacial deformity on the chance of being hired for a job

**DOI:** 10.3389/fpsyg.2023.1037167

**Published:** 2023-12-01

**Authors:** Bruna Marlene de Miranda, Patricia Tavian Gonçalez Miguel, Flavio Magno Gonçalves, Davani Latarullo Costa, Thalita de Paris Matos Bronholo, Odilon Guariza-Filho, José Stechman-Neto, Rosane Sampaio Santos, Bianca Simone Zeigelboim, Cristiano Miranda de Araujo

**Affiliations:** ^1^School of Dentistry, Tuiuti University of Paraná, Curitiba, PR, Brazil; ^2^Postgraduate Program in Human Communication Health, Tuiuti University of Paraná, Curitiba, Brazil; ^3^Oral and Maxillofacial Surgery, ILAPEO, Curitiba, Brazil; ^4^Department of Orthodontics, Pontifícia Universidade Católica do Paraná, Curitiba, Brazil

**Keywords:** job application, dentofacial deformities, orthognathic surgery, esthetics, malocclusion

## Abstract

**Objective:**

This study aimed to determine the impact of dentofacial deformity on an individual’s chances of being hired for a hypothetical job involving customer service.

**Materials and methods:**

Face photographs (frontal and lateral) of 15 patients with moderate to severe dentofacial deformity, taken before and after orthodontic-surgical correction, were selected and randomized between two different questionnaires. In addition, five patients without dentofacial deformity were used as controls in both questionnaires. These questionnaires were taken by adults responsible for hiring personnel to work in commerce and business activities, graduates or postgraduates in business administration, with experience in recruiting and hiring personnel. The evaluation took place using a Likert scale with values ranging from 0 to 10 (in which 0 corresponded to complete disagreement and 10 to complete agreement), considering the following variables in a first impression judgment: honesty, intelligence, productivity at work, and hiring chance. Data were tabulated and statistical analysis was performed using a linear regression model for the explanatory variables that showed statistical significance in the analysis of variance (ANOVA). Effect size through Cohen’s d has been corrected for all comparisons performed.

**Results:**

All re-examined domains demonstrated statistical differences even when included in a multivariate model (*p* < 0.05), with lower mean values for those requiring pre-treatment (presenting deformity), although the effect size was small for all comparisons.

**Conclusion:**

Dentofacial deformity influenced the hiring chance, although not appearing to be a preponderant factor for hiring, acting as a tiebreaker among the candidates adopted.

## 1 Introduction

Facial features have been of great interest in several areas of knowledge due to the ability of human beings to process, recognize, and extract information from other people’s faces (Little, 2014), playing an important role in social life. In this context, the smile is frequently the first characteristic to be noticed and evaluated (Didier et al., 2019; Almedlej et al., 2020). Individuals who have dentofacial disharmony tend to have impaired social acceptance, and society’s considerations regarding this individual tend to be less positive (Davis et al., 1998; Henson et al., 2011; Yu et al., 2014; Kämäräinen et al., 2019; Almedlej et al., 2020; Saghafi et al., 2020). These individuals also tend to be perceived as being less intelligent and less likely to be hired in hypothetical job interviews due to the presence of malocclusion (Almedlej et al., 2020).

On the other hand, more attractive people are noticed as more competent in job interviews and are also thought to have the right to own higher positions and salaries (Almedlej et al., 2020), being seen in a more positive and effective way (Lorenzo et al., 2010) and inducing people around them to behave more honestly (Wang et al., 2017). Having a harmonious face and an ideal smile result in greater self-confidence and social acceptance, better educational potential, and other qualities, such as also appearing to have a higher intellectual level (Shaw et al., 1985; Davis et al., 1998; Henson et al., 2011; Kolawole et al., 2012; Ng et al., 2013; Yu et al., 2014; Didier et al., 2019; Kämäräinen et al., 2019; Posnick et al., 2019; Almedlej et al., 2020; Saghafi et al., 2020).

The orthodontic-surgical treatment, when successful, corrects the dentofacial deformity, improving the aesthetic, functional, social, and psychological aspects (De Araujo et al., 2020). Facial cosmetic surgery can improve the first impression an individual creates, making this individual to be rated after surgery as more attractive, better in social skills, more successful in dating, better in athletic skills, and more financially successful (Dayan et al., 2004). It is expected that with the improvement of facial harmony, many aspects of life would improve, including the professional career (Baldwin, 1980; Ng et al., 2013; Yu et al., 2014; Kämäräinen et al., 2019; Posnick et al., 2019; Almedlej et al., 2020; Saghafi et al., 2020). The changes promoted by orthognathic surgery can influence both how the individual is viewed by other people and their own self-perception. The correction of dentofacial deformity can promote improvement in several psychological domains, mainly in aspects related to depression (Sebastiani et al., 2021; Basso et al., 2022).

Considering the implications that the lack of facial esthetics can generate on other people’s judgment and the benefit that orthodontic-surgical treatment can provide, the present study aimed to evaluate the association between the presence/absence of dentofacial deformity in relation to the following aspects: chance of being hired for a hypothetical job vacancy involving customer service and first impression judgment regarding honesty, intelligence, and productivity at work.

## 2 Materials and methods

This study was approved by the ethics committee of Tuiuti University of Paraná (approved no. 4.353.486). To ensure transparency and avoid the possibility of occurrence of bias in the selective reporting of outcomes, this observational cross-sectional study was conducted and reported using the guideline “Strengthening the Reporting of Observational Studies in Epidemiology (STROBE)” Statement (von Elm et al., 2008), being registered on the OpenScience Framework platform (https://osf.io) before its beginning (doi: 10.17605/OSF.IO/GMDRU).

### 2.1 Context

The survey was conducted offsite through self-administered questionnaires sent through a digital platform (Google Forms - Google Corp, Mountain View, California, EUA).

**Figure 1:**
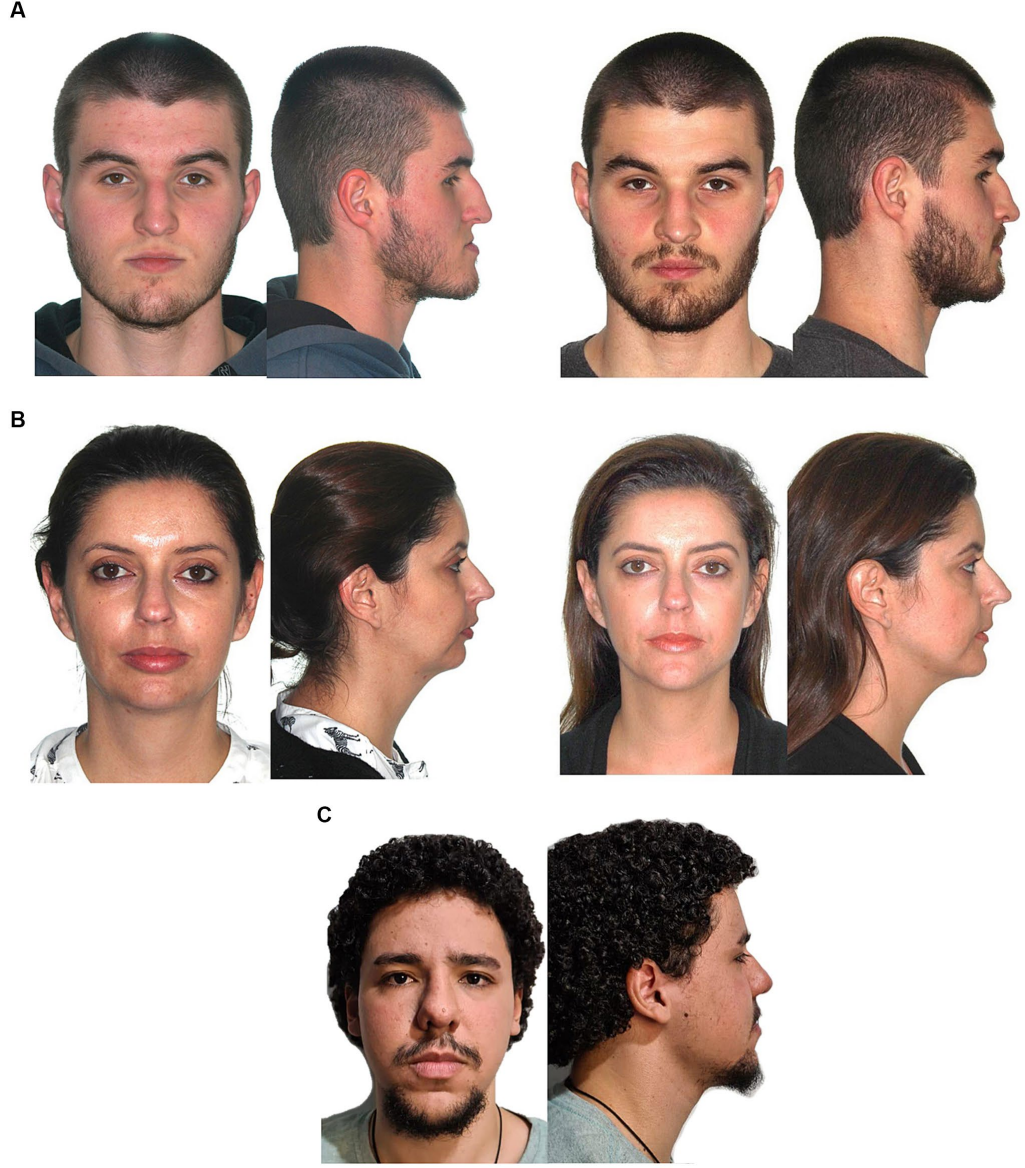
Images of the subjects: **(A)** Dentofacial deformity due to the presence of a preoperative concave profile (questionnaire 1) and the postoperative outcome (questionnaire 2); **(B)** Dentofacial deformity due to the presence of a preoperative convex profile (questionnaire 2) and the postoperative outcome (questionnaire 1); **(C)** Control patient with a straight facial profile and absence of dentofacial deformity (questionnaires 1 and 2).

To assess the impact of the presence/absence of dentofacial deformity in a judgment on a first impression, two complementary questionnaires were constructed with the respective evaluation domains (hire chances, honesty, intelligence, and productivity) to be judged using a Likert scale. The questionnaires consisted of facial photographs (front and side) of 15 people taken before (T1) or after orthodontic-surgical correction of the dentofacial deformity (T2). When the photograph in T1 of one these people was allocated to one questionnaire, the photograph in T2 of the same individual was automatically allocated to the other questionnaire. The position of each individual was maintained between the questionnaires, changing only the presence/absence of the deformity. The photographs of the 15 individuals who composed the questionnaire were selected by two authors, and they had to present malocclusion with moderate to severe impairment of the facial profile with surgical indication for correction of Angle Class II or III malocclusion on T1 and post-operative photographs, after orthodontic-surgical correction. Vertical intra-group discrepancies were not considered. To ensure the proper pairing of comparisons, the preoperative photographs of each individual were randomly divided, using a computational method, between two questionnaires with the same equal probabilities of allocation, and in a complementary way, the postoperative photographs were also distributed between the questionnaires. In addition, photographs of five control patients (without dentofacial deformity) were included in both questionnaires. Thus, each questionnaire at the end of randomization presented a mixture of Class II and Class III patients, treated and untreated through orthodontic-surgical correction, and the same control patients (without dentofacial deformity), totaling 20 photographs per questionnaire. Consent forms were collected from all patients included in the questionnaires. Examples of the photographs used in the T1, T2, and the control are shown in Figure 1.

### 2.2 Participants

The questionnaires were applied to people who met the following eligibility criteria: adults, aged over 18, responsible for hiring employees to work in commerce and business activities, and with experience in the recruiting and hiring process. Individuals who were not employed as hiring personnel were excluded from the sample. Informed consent forms were collected from all individuals included in the sample.

### 2.3 Variables, data sources, and measurement

The questionnaires were also randomly divided among the respondents, with each evaluator being asked to judge the photographs contained in the questionnaire (hypothetical candidates for a job vacancy) taking into account the hypothetical scenario of the need to hire 10 people for customer service in their company. For this, a Likert scale with values ranging from 0 to 10 (where 0 corresponds to complete disagreement and 10 to complete agreement) was used to judge first impressions regarding honesty, intelligence, productivity at work, and the chance of hiring. At no point were the participants informed that the study was related to dentofacial deformity. The mean scores of each assessment domain were considered dependent variables for comparison between groups (pre- and postoperative periods and control). The evaluation time (pre-surgical, post-surgical and control), the initial malocclusion (Angle’s Class I, II, and III), age and gender (patient and evaluator) were considered independent variables.

### 2.4 BIAS

To ensure a greater representation of the study population, individuals with deformities ranging from moderate to severe were included, provided that they had surgical indication for their correction. The inclusion of the same control group between both questionnaires enabled to evaluate whether the judgments could be compared. In addition, a multivariate linear regression model was conducted to adjust estimates for the independent variables.

### 2.5 Study size

To ensure the statistical representativeness of the sample, a pilot study with 20 evaluators was carried out, enabling to calculate the adequate number of respondents to comprise the sample, taking into account a statistical power of 80% and a significance level of 5%, alpha = 0.05. Through the pilot study, an effect size of 0.2 (Cohen’s *d*) was determined, requiring a number of 262 evaluations for each group.

### 2.6 Statistical analysis

To provide greater confidence, the researcher responsible for data analysis was blinded, and the data of each group were coded prior to comparisons. Data normality was tested using the Kolmogorov–Smirnov test, while homoscedasticity was tested using the Levene test. As the data were normally distributed and the questionnaires were independently evaluated (not paired), one-way analysis of variance (ANOVA) was performed considering the difference between the mean scores obtained for each domain and between the different groups (pre- and postoperative periods and control). When ANOVA indicated a significant difference, the Tukey’s test was used to compare means. The variables that presented statistical significance (*α* = 5%) were included in a multivariate linear regression model, estimating adjusted β coefficients for each variable, with the respective standard errors. To assess the reliability of the responses obtained between evaluators, the Student t test was also used to compare the scores of the two forms among the control group. All analyzes were performed using the statistical software Jasp., version 0.14.1 and SPSS, version 16.0 (IBM SPSS, Armonk, NY), adopting a significance level of 5%. To assess the effect size observed between the mean differences, Cohen’s d was used.

## 3 Results

A total of 56 evaluators were included; one of them was excluded for not meeting the eligibility criteria, thus totaling 55 evaluators who judged the 20 patients in each questionnaire (405 evaluations for the preoperative period, 420 for the postoperative period, and 275 for the control group). One of the questionnaires had one more evaluator than the other, generating an inequality of 15 evaluations between the pre and postoperative period.

There was a significant difference for all domains evaluated, when considering the evaluation time (Table 1), with higher mean values observed between the control and postoperative groups. The preoperative group presented the lowest mean values for all domains evaluated, being judged as less likely to be hired, less honest, less intelligent, and less productive (*p* < 0.01) than individuals with good facial harmony (operated or not). Individuals with good facial harmony but who were not operated were the ones with the greatest chance of being hired (*p* < 0.05). When considering the initial malocclusion, patients with Class III malocclusion presented the worst judgment when compared to the others (*p* < 0.05). There was no difference when considering the gender of the patient or the evaluator (*p* > 0.05). The variables evaluation time, initial malocclusion and age were included in a multivariate model, maintaining the statistical significance observed in the initial analyses. Age showed an inverse relationship with the chance of being hired and the judgment regarding productivity (*p* < 0.05) (Table 2).

**Table 1:** Comparisons of the average scores obtained considering the evaluation moment, malocclusion and gender for the different assessment domains.

Explanatory variable	Category	Hiring		Honesty		Intelligence		Productivity	
		Mean (SD)	value of *p*	Mean (SD)	value of *p*	Mean (SD)	value of *p*	Mean (SD)	value of *p*
Patient’s gender	Male	7.01 ± 2.24^A^	0.941	7.10 ± 1.97^A^	0.154	7.07 ± 1.91^A^	0.534	6.83 ± 2.02^A^	0.875
	Female	7.02 ± 2.37^A^		7.27 ± 1.88^A^		6.99 ± 1.99^A^		6.85 ± 2.16^A^	
Evaluator’s gender	Male Female	7.06 ± 2.26^A^ 6.98 ± 2.36^A^	0.596	7.25 ± 1.90^A^ 7.17 ± 1.94^A^	0.483	7.06 ± 1.95 6.98 ± 1.97	0.502	6.92 ± 2.02 6.77 ± 2.18	0.239
Malocclusion	Class I	7.55 ± 2.04^A^	<0.001	7.41 ± 1.73^A^	<0.001	7.47 ± 1.76^A^	<0.001	7.31 ± 1.90^A^	<0.001
	Class II	7.04 ± 2.25^B^		7.31 ± 1.77^A^		7.05 ± 1.83^B^		6.88 ± 2.07^B^	
	Class III	6.44 ± 2.56^C^		6.80 ± 2.28^B^		6.51 ± 2.26^C^		6.33 ± 2.24^C^	
Evaluation moment	Pre-surgery	6.64 ± 2.42^A^	<0.001	6.96 ± 2.08^A^	0.005	6.60 ± 2.14^A^	<0.001	6.42 ± 2.22^A^	<0.001
	Post-surgery	7.04 ± 2.32^B^		7.31 ± 1.85^B^		7.13 ± 1.82^B^		6.96 ± 2.04^B^	
	Control	7.55 ± 2.04^C^		7.41 ± 1.73^B^		7.47 ± 1.76^B^		7.31 ± 1.90^A^	

*Value of *p* of the ANOVA test (significance level 5%).

Different letters on the same line indicate statistical significance.

**Table 2:** Explanatory variables of the scores obtained for each assessment domain.

Domain	Explanatory variable	*β*		Standard error	Value of p*
		Non-standard	Standardized		
Hiring	Intercept	7.001	–	0.307	<0.001
	Evaluation moment	0.396	0.167	0.163	0.016
	Initial malocclusion	0.809	0.341	0.191	<0.001
	Age	−0.031	−0.107	0.011	0.005
Honesty	Intercept	6.651	–	0.134	<0.001
	Evaluation moment	0.317	0.161	0.136	0.02
	Initial malocclusion	0.488	0.247	0.145	<0.001
Intelligence	Intercept	6.274	-	0.135	<0.001
	Evaluation moment	0.501	0.250	0.138	<0.001
	Initial malocclusion	0.513	0.256	0.146	<0.001
Productivity	Intercept	6.625	–	0.277	<0.001
	Evaluation moment	0.536	0.250	0.147	<0.001
	Initial malocclusion	0.685	0.319	0.172	<0.001
	Age	−0.022	−0.086	0.009	0.023

^*^Value of p obtained by the multivariate linear regression model.

For all comparisons performed, the effect size assessed by Cohen’s d was considered small, while the statistical power of the test was >80%. There was no statistical difference between the two questionnaires applied when considering the comparison between the control group (p > 0.05).

## 4 Discussion

Physical appearance influences a person’s self-esteem, the way this person behaves and socializes, and consequently also intervenes in society’s considerations regarding this individual. A harmonious face and a smile considered beautiful are factors of great value for social care (Didier et al., 2019; Almedlej et al., 2020). People with malocclusions and thus presenting a disharmony of the face receive more negative claims regarding their character and intelligence when compared to people with harmonious faces (Almedlej et al., 2020). In addition, surgical orthodontic correction can generate changes in the lifestyle of these individuals, positively influencing their personal and social relationships (Basso et al., 2022). Thus, the present study evaluated whether the presence of dentofacial deformity could influence the chance of being hired for a job, besides conducting a first impression assessment of honesty, intelligence, and productivity at work. It was possible to observe that the dentofacial deformity influenced these variables negatively, although not absolutely, for hiring the evaluated individual.

First impressions are instinctively formed based on facial appearance and can occur very quickly, even from the first contact with an individual (Bar et al., 2006; Posnick and Kinard, 2019). Smile aesthetics also play an important role in job seeking. According to Pithon et al. (2014), when evaluating the influence of dental aesthetics in the search for a job, people with ideal smiles were considered more intelligent and had a greater chance of being hired when compared to people with non-ideal smiles. On the other hand, individuals undergoing orthodontic-surgical treatment to correct mandibular deficiency are perceived as more dominant, reliable, intelligent, attractive, friendly, and less threatening (Posnick and Kinard, 2019). The present study did not take into account photographs of smiles, thus enabling the assessment of the patients’ facial profiles and of whether this could influence hiring chances in a hypothetical job opening for the public.

Other authors evaluated influences from the dental field on employment hiring. Didier et al. (2019) analyzed the influence of orthodontic appliances on the perceptions of individuals responsible for hiring during a job interview by comparing photographs of smiling individuals with different orthodontic appliance designs. The results showed that more esthetic appliances had a higher acceptance rate for recruitment according to evaluators, showing that the better the esthetics of the orthodontic appliance, the greater the probability of being hired. The results of the study aforementioned corroborate the data obtained in this study, in which dentofacial esthetics collaborated for a possible hiring. Additionally, the evaluation of the attractiveness of the face is altered according to the different types of malocclusions, with only the total width of the face, labial area, upper labial, and mento labial angles, which appear to influence all malocclusions (Parul et al., 2022). Thus, in the present study, the different types of deformity, considering anteroposterior deviations, were included in the analysis.

When considering the assessment of honesty and effectiveness at work by persons responsible for hiring individuals regarding the impact of dental appearance, Almedlej et al. (2020) observed that both variables did not show a significant difference. Similarly, Pithon et al. (2014) found no difference when considering the honesty and effectiveness of these individuals, demonstrating that issues related to character and ability may not be evaluated through an image. On the other hand, when considering the aesthetics of the face, according to Rankin and Borah (2003), individuals who have facial aspects considered normal and physically attractive are seen as significantly more positive in many social, professional, and psychological spheres, while individuals who have scars and skin deformities involving the face are perceived as having impaired social functionality, employability, honesty, reliability, and effectiveness. These data corroborate the present findings, in which honesty and productivity showed different results when comparing preoperative, postoperative, and control patients, with control and postoperative patients being better evaluated in these aspects.

The orthodontic-surgical treatment is dependent on knowledge and management strategies between the surgeon and orthodontist to provide the best esthetic results for the patient. Both the performance of the orthodontist and the surgeon, even if isolated, can compromise the result positively or negatively (Wolford, 2020). Dependence on good teamwork increases the probability of error and even influences the risk of late relapse. In the present study, the control group composed of individuals who did not undergo orthognathic surgery, and who had good facial harmony, had a greater chance of being hired and proved to be more productive, compared to operated patients. The effect size for this comparison was considered small. The hypothesis to explain this difference is that even with a significant difference between the pre and postoperative periods, subtle differences in the face of individuals with good facial harmony may exist, generating changes in the judgment of the first impression. More studies focused on the comparison between operated individuals and those with good facial harmony should be carried out, to address this difference.

Some limitations of the present study should be pointed out, such as the assessment using two-dimensional images, the assessment focused only on the facial aspect, and also confounding factors inherent to any observational study. Unevaluated factors, such as skin characteristics, individual personality and voice, can impact the initial judgment and chance of hiring, which is one of the limitations of the study. In addition, the static evaluation of the face can alter the judgment when compared to the same face with movement. On the other hand, knowledge on the influence of dentofacial deformities on employability demonstrates the importance of this type of treatment in the social aspect, going further just aesthetic and functional correction.

In conclusion, based on our findings people with dentofacial deformities are perceived as impaired when looking for a job compared to individuals with good facial harmony (operated or not) both in terms of chances of being hired and in aspects related to honesty, intelligence, and productivity. However, this impaired perception is not preponderant in their hiring due to the small effect size observed, being considered an evaluation item that can serve as a tiebreaker in a hypothetical vacancy.

## Data availability statement

The original contributions presented in the study are included in the article/supplementary material, further inquiries can be directed to the corresponding author/s.

## Ethics statement

The studies involving human participants were reviewed and approved by the Ethics committee of Tuiuti University of Paraná. The patients/participants provided their written informed consent to participate in this study. Written informed consent was obtained from the individual(s) for the publication of any potentially identifiable images or data included in this article.

## Author contributions

BM, PG,FG, DC, and CA conceptualized and designed the study. FG organized the database. JS-N and CA performed the statistical analysis. BM, RS, and BZ wrote the first draft of the manuscript. OG-F, TB, and JS-N wrote the sections of the manuscript. All authors contributed to manuscript revision, read, and approved the submitted version.
